# The Impact of SMAD4 Loss on Outcome in Patients with Advanced Pancreatic Cancer Treated with Systemic Chemotherapy

**DOI:** 10.3390/ijms18051094

**Published:** 2017-05-19

**Authors:** Steffen Ormanns, Michael Haas, Anna Remold, Stephan Kruger, Stefan Holdenrieder, Thomas Kirchner, Volker Heinemann, Stefan Boeck

**Affiliations:** 1 Institute of Pathology, Ludwig-Maximilians Universität München, Thalkirchner Str. 36, 80337 Munich, Germany; annaremold@yahoo.de (A.R.); thomas.kirchner@med.uni-muenchen.de (T.K.); 2 Department of Internal Medicine III and Comprehensive Cancer Center, Klinikum Grosshadern, Ludwig-Maximilians Universität München, Marchioninistr. 15, 81377 Munich, Germany; michael.haas@med.uni-muenchen.de (M.H.); stephan.kruger@med.uni-muenchen.de (S.K.); volker.heinemann@med.uni-muenchen.de (V.H.); 3 Institute of Laboratory Medicine, German Heart Centre Munich, Technische Universität München, 80333 Munich, Germany; holdenrieder@dhm.mhn.de; 4 Institute of Clinical Chemistry and Clinical Pharmacology, Universitätsklinikum Bonn, 53127 Bonn, Germany; 5 Deutsches Konsortium für Translationale Krebsforschung (DKTK, German Cancer Consortium), German Cancer Research Center (DKFZ), 69120 Heidelberg, Germany

**Keywords:** advanced pancreatic cancer, SMAD4, DPC4

## Abstract

The role of the tumor suppressor mothers against decapentaplegic homolog 4 (SMAD4) has not yet been defined in patients (pts) with advanced pancreatic cancer (aPC). This translational research study was designed to evaluate the impact of tumoral SMAD4 loss on clinicopathological parameters and outcome in PC patients receiving palliative chemotherapy. Using immunohistochemistry, we examined SMAD4 expression in tumor tissue of 143 aPC pts treated within completed prospective clinical and biomarker trials. In uni- and multivariate analyses, SMAD4 expression status was correlated to clinicopathological patient characteristics and outcome. At chemotherapy initiation, 128 pts had metastatic PC; most pts (*n* = 99) received a gemcitabine-based regimen. SMAD4 loss was detected in 92 pts (64%); patient characteristics such as gender, age, tumor grading, disease stage or number of metastatic sites had no significant impact on tumoral SMAD4 status. In univariate analyses, SMAD4 loss had no impact on overall survival (hazard ratio (HR) 1.008, *p* = 0.656); however, we observed a prolonged progression-free survival (HR 1.565, *p* = 0.038) in pts with tumoral SMAD4 loss. This finding was confirmed in multivariate analyses (HR 1.790, *p* = 0.040), but only for gemcitabine-treated pts. In contrast to previous studies in resectable PC, loss of SMAD4 expression was not associated with a negative outcome in patients with advanced PC receiving systemic chemotherapy.

## 1. Introduction

Pancreatic cancer (PC) remains a highly malignant disease with a dismal prognosis; current projections expect an increase in annual PC deaths in the US from 36,700 in 2010 to 63,000 in 2030, making PC the second leading cause of cancer death in the US [1]. Progress in PC research has been steady but slow during the last decades, with significant steps forward made specifically in improving adjuvant and systemic treatment options. For patients with metastatic disease, the introduction of 5-FU, folinic acid, irinotecan, oxaliplatin (FOLFIRINOX), nanoparticle albumin-bound paclitaxel (nab-paclitaxel) and more recently nanoliposomal irinotecan has led to better results regarding survival and also quality of life [2,3]. However, validated prognostic and—even more important—predictive biomarkers (for the efficacy of a specific treatment regimen) are still lacking in this disease [4].

The tumor suppressor mothers against decapentaplegic homolog 4 (SMAD4) (also known as deleted in pancreatic cancer 4 (DPC4)) was first described in 1996 by Hahn and co-workers and is thought to regulate pancreatic cell proliferation and apoptosis via the transforming growth factor-β (TGF-β) pathway [5,6]. A loss of SMAD4 expression is observed in about half of all patients diagnosed with PC and it has been hypothesized that SMAD4 inactivation is associated with a worse prognosis and with the pattern of disease recurrence/metastatic progression in localised PC [6,7,8]. In the search of prognostic biomarkers that could, e.g., allow a better treatment stratification, the role of the tumor suppressor SMAD4 has already been investigated in several previous studies in PC. In those investigations, SMAD4 was mainly analyzed in patients with resectable disease, most likely because tumor tissue is easily available in such patients [7,8,9,10,11,12]. However, results from previous studies are inconclusive: most of these analyses were performed as retrospective studies and found a negative prognostic impact of a loss in SMAD4 on survival [10,11]. A single-center series from Memorial Sloan-Kettering Cancer Center in contrast could not confirm previous observations and reported that a loss of SMAD4 was neither associated with a metastatic recurrence pattern nor with early death in resected PC [12].

Two recent meta-analyses reported that a loss of SMAD4 expression in PC was significantly correlated with an inferior overall survival (OS) (hazard ratio (HR) 1.20, 95% confidence interval (CI) 1.03–1.40 and HR 0.61, 95% CI 0.38–0.99, respectively) and that the frequency of SMAD4 protein loss was significantly increased in pancreatic ductal adenocarcinoma than in nonmalignant pancreatic tissue, making it also a potential diagnostic marker for PC [13,14].

We performed a retrospective translational study that included tumor tissue from patients with advanced PC only, obtained from completed German multicenter clinical trials as well as from a prospective single center biomarker study conducted at our comprehensive cancer center. SMAD4 expression was analyzed centrally by immunohistochemistry (IHC) and biomarker data were correlated with clinical parameters as well as outcome (e.g., progression-free survival (PFS) and OS).

## 2. Results

### 2.1. Patient Characteristics and Clinicopathological Variables

Median age within the study population (84 male, 59 female) was 63 years. Data on Karnofsky performance status (KPS) was available from 133 patients (pts) only. Seventy-six pts had a KPS >80%, which was associated with an improved OS (median 11.1 vs. 6.3 months, *p* = 0.017, Figure S1A), but not with PFS (median 7.6 vs. 4.1 months, *p* = 0.056; Figure S1B, Table 1). Only 15 of the 143 patients had locally advanced pancreatic cancer (LAPC) and 128 patients had metastatic disease at chemotherapy initiation, with the latter being associated with shorter OS and PFS from the beginning of palliative chemotherapy (OS 13.6 vs. 8.1 months, *p* = 0.064; PFS 10.0 vs. 6.3, *p* = 0.054), though not statistically significant (potentially due to the smaller number of LAPC patients). Ninety-nine patients received a gemcitabine-based first-line treatment, 25 patients received fluoropyrimidine-based chemotherapy and 19 patients received a gemcitabine- and fluoropyrimidine-containing regimen, with no significant differences in OS respectively (OS 8.3 vs. 9.1 vs. 11.0 months, *p* = 0.250; Figure S1C, Table 1). However, we detected significant differences in PFS according to the type of first-line chemotherapy applied (7.6 vs. 4.0 vs. 4.7 months, *p* = 0.017, Figure S1D, Table 1). The 15 LAPC patients by definition had no clinically apparent metastasis upon therapy initiation, 90 patients had a single metastatic site and 38 patients had more than one metastatic site, which was not associated with a shorter OS or PFS (OS 13.6 vs. 8.3 vs. 7.8 months, *p* = 0.180; PFS 10.0 vs. 6.6 vs. 4.7 months, *p* = 0.097). Median OS calculated from the initiation of palliative chemotherapy of the entire study cohort was 8.3 months (95% CI 6.845–9.779) and median PFS was 6.6 months (95% CI 5.574–7.634). Neither patient age group (<60 years vs. ≥60 years), nor sex or tumor grade were associated with OS or PFS. All clinicopathological patient characteristics and associated OS and PFS times are summarized in Table 1.

### 2.2. Mothers against Decapentaplegic Homolog 4 (SMAD4) Expression, Clinicopathological Variables and Survival Analyses

Eighty samples (55.9%) were biopsy material and 63 samples were derived from surgical resections (44.1%). We detected similar rates of SMAD4 loss in biopsy material (50 of 80, 62.5%) and resection material (42 of 63, 66.7%). Seventy-two samples (50.3%) represented metastatic tissue and 71 samples contained the primary tumor (49.7%). In the metastatic samples, SMAD4 expression was lost in 43 cases (59.7%) whereas in the primary tumor samples SMAD4 loss was detected in slightly higher rates (49 of 71 cases, 69.0%), a difference that was not statistically significant (*p* = 0.162). Equally, as summarized in Table S1, no statistically significant correlation of SMAD4 expression with age group, gender, KPS, stage of disease at start of palliative chemotherapy, tumor grading and number of metastatic sites was detected using cross tabulations.

**Figure 1:**
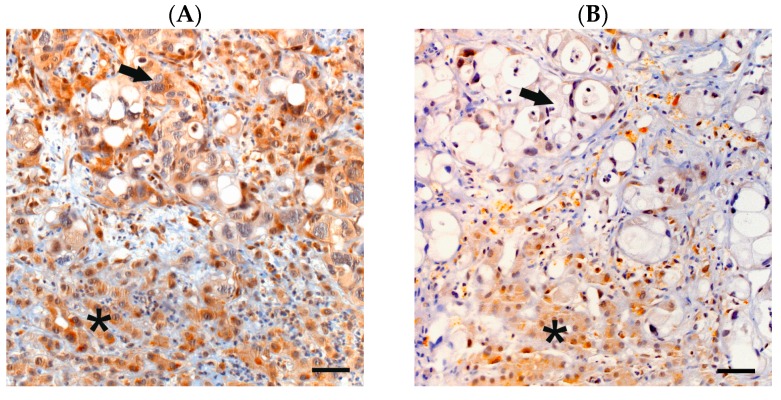
Lost and preserved mothers against decapentaplegic homolog 4 (SMAD4) expression in pancreatic cancer (PC) tissue. Immunohistochemical staining of SMAD4 in exemplary PC samples shows preserved (**A**) as well as lost SMAD4 expression (**B**) in the tumor tissue (arrow) whereas SMAD4 expression is preserved in adjacent liver parenchyma (asterisk). 200× magnification; scale bars indicate 50 µm.

**Figure 2:**
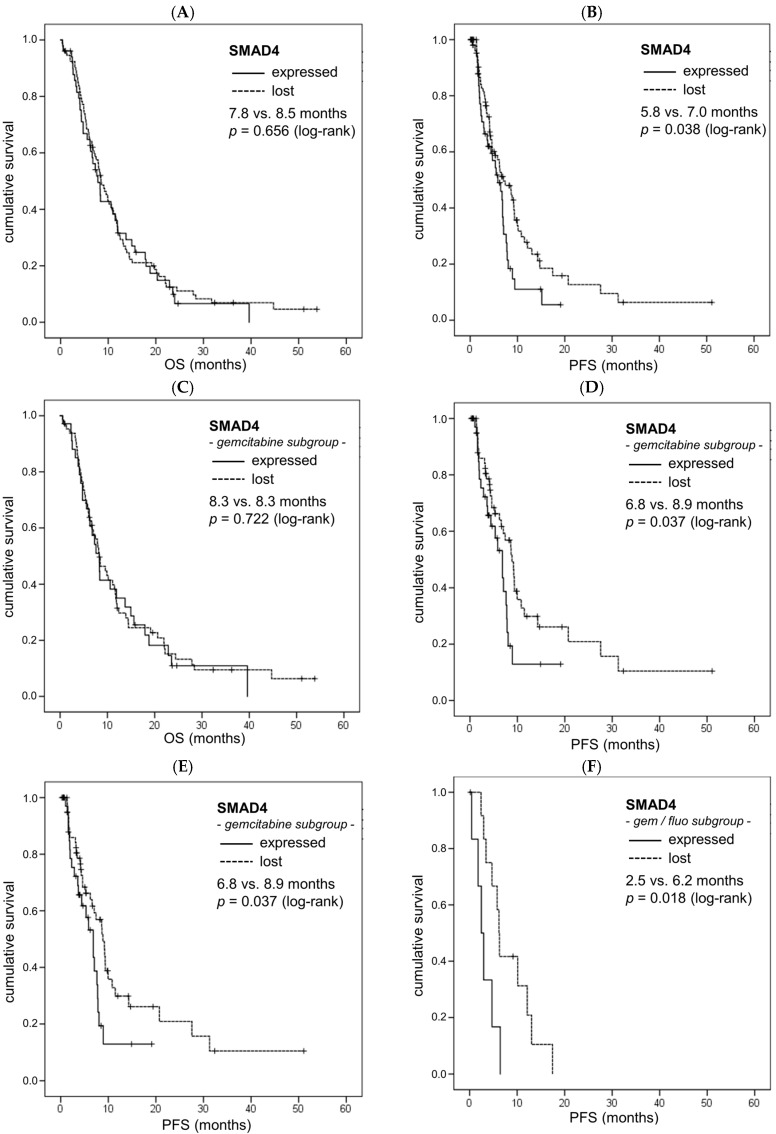
Loss of SMAD4 expression correlates with improved progression-free survival (PFS) but not overall survival (OS) in advanced PC (aPC), especially in gemcitabine-treated patients (pts). Univariate analysis (Kaplan–Meier curves and log-rank tests) of SMAD4 expression and OS as well as PFS in the total study population (**A**,**B**), the gemcitabine-based treatment subgroup (**C**,**D**) and the gemcitabine–fluoropyrimidine-based treatment subgroup (**E**,**F**) as a biomarker in the tumor tissue of palliatively-treated aPC pts. Crossed lines indicate censored cases.

We examined SMAD4 expression in tumoral tissue and adjacent normal tissue, i.e., pancreatic parenchyma/stroma or adjacent normal tissue of the homing organ in the case of metastatic samples (Figure 1). SMAD4 expression was lost in 64% of the tumors in the overall study population and in similar rates in each chemotherapy subgroup (Table 2). In the overall study population, a loss of SMAD4 expression was not associated to OS (median 8.5 vs. 7.8 months, *p* = 0.656; Figure 2A, Table 2). However, patients showing a loss of SMAD4 expression in their tumor tissue had a statistically significantly longer PFS than patients with preserved SMAD4 expression (median 7.0 vs. 5.8 months, *p* = 0.038, Figure 2B, Table 2).

Similarly, in the patient subgroup treated with gemcitabine-based chemotherapy (*n* = 99), no significant association of SMAD4 expression with OS but a significantly longer PFS was detected when SMAD4 was lost (median OS: 8.3 vs. 8.3 months, *p* = 0.722, Figure 2C; median PFS: 8.9 vs. 6.8 months, *p* = 0.037, Figure 2D, Table 2). In the small patient subgroup treated with fluoropyrimidine-based chemotherapy (*n* = 25), we found no significant impact of SMAD4 expression on OS or PFS (median OS: 11.5 vs. 7.2 months, *p* = 0.104; median PFS: 6.7 vs. 3.6 months, *p* = 0.185, Table 2). In the even smaller subgroup of patients treated with gemcitabine- and fluoropyrimidine-containing chemotherapy (*n* = 19), we detected a significant positive correlation of SMAD4 loss with an improved OS as well as PFS (median OS: 12.5 vs. 4.3 months, *p* = 0.017, Figure 2E; median PFS: 6.2 vs. 2.5 months, *p* = 0.018, Figure 2F, Table 2).

In stepwise forward multivariate Cox regression analyses, adjusting for KPS group, chemotherapy type, SMAD4 expression and disease stage at start of palliative chemotherapy, KPS group was the only independent prognostic factor for OS in the overall patient population (*p* = 0.018, HR 1.570, 95% CI 1.081–2.280), whereas no independent prognostic factors for PFS were detected. Interestingly, within that analysis, SMAD4 expression had an independent impact on PFS in the gemcitabine-treated subgroup (*p* = 0.040, HR 1.790, 95% CI 1.028–3.116).

## 3. Discussion

To the best of our knowledge, this is the first study examining SMAD4 expression in palliatively-treated patients with advanced PC besides a study on rapid autopsy samples [7]. Using IHC on resection tissue and biopsy samples from primary tumors and metastatic lesions, we were able to show that a loss of SMAD4 expression occurs in a slight majority of the tumors (overall 64%). Importantly, we found similar rates of SMAD4 loss independent of the tissues’ origin, i.e., pancreatic primary or metastasis. Although we could not compare primary tumors and metastatic tissue of the same pts within our present analysis, our finding is supported by previous observations detecting no difference in SMAD4 status between pancreatic primary and corresponding metastases [7]. In contrast to previously published studies on SMAD4 as a prognostic biomarker in resected PC [10,11,15,16,17,18], we did not detect a negative prognostic impact of SMAD4 loss on OS or PFS in our study population. In fact, patients showing a loss of SMAD4 in their respective tumor tissue had a significantly better PFS than patients with SMAD4-positive tumors, most notably in the gemcitabine-treated subgroup where the SMAD4 status actually was an independent prognostic factor for PFS. The reported ratio of SMAD4 loss in our study is slightly higher than reported in most studies of SMAD4 expression in resected PC, which was ranging between 50% and 60% [9,10,15,19,20]. However, the previously reported rates of SMAD4 loss had a broad range between 32% [12,21] and 81.6% [16]. This could be due to the high heterogeneity in the applied detection methods (different antibodies and IHC methods) but could also be due to the differences in scoring algorithms and corresponding cut-offs to categorize a tumor as “SMAD4 lost” or “SMAD4 expressed”. In the context of a recent study [22], this heterogeneity in detection and the lack of ability to detect small changes in SMAD4 expression, such as in *SMAD4* gene haploinsufficiency, may become crucial. Using genetically defined mouse models of pancreatic cancer in a *KRAS* and *TP53* mutant background, Whittle et al. showed that a heterozygous deletion of *SMAD4* leads to locally aggressive disease when tumoral expression levels of runt-related transcription factor 3 (RUNX3) are low, but to extensive metastatic spread when RUNX3 levels are high, similarly as in the situation of *SMAD4* homozygous deletion [22]. Thus, as a biomarker for clinical decision making, differential SMAD4 expression possibly needs to be interpreted in the context of RUNX3 expression. Moreover, even if the ratios of SMAD4 loss may be similar in each single study, the majority of studies claiming SMAD4 loss as a strong negative prognostic biomarker were carried out in resected PC of primarily Asian populations [10,15,16,17], whereas some equally large studies in European, American or Australian populations did not detect this association [9,12,19,20]. This may perhaps also point towards a potential interaction of SMAD4 loss and the patients’ genetic background, although a high heterogeneity in treatment modalities and standards of care may contribute to this observation as well.

In terms of subgroup interpretation according to the chemotherapy regimen used, we found a significant association of SMAD4 loss to better PFS in gemcitabine-based treated patients and to improved OS as well as PFS in patients that received both gemcitabine- and fluoropyrimidine-based chemotherapy. However, the latter subgroup was very small (*n* = 19). Consequently, these findings should be interpreted very carefully towards a potential predictive value of SMAD4 loss in gemcitabine or gemcitabine–fluoropyrimidine-treated advanced PC patients, although they are in line with previously published data showing a benefit from adjuvant gemcitabine treatment in SMAD4-negative tumors only [9]. This observation could be due to a higher sensitivity to gemcitabine treatment as SMAD4-negative tumor cells show increased proliferation [23]. However, SMAD4 negative cells even showed higher resistance to gemcitabine treatment than their SMAD4 positive counterparts [24].

Its retrospective nature, the heterogeneous chemotherapy regimens applied, as well as the use of both biopsy material and resection specimens can be considered as the main limitations of our present study. However, these factors may partially also be seen as the studies´ strength, as our findings represent a broad range of clinically variable patients with advanced PC, which make up the vast majority of PC patients in daily clinical practice.

## 4. Materials and Methods

### 4.1. Patient Population

Tumor samples of 143 patients with advanced PC were collected retrospectively from patients treated within terminated German multicenter chemotherapy trials as reported previously [25]. Clinical data were retrieved from the participating hospitals and the studies’ databases. Only patients receiving conventional cytotoxic chemotherapy were included, excluding patients treated with targeted agents such as erlotinib. OS times were calculated from the start of palliative first-line chemotherapy to death from any cause. According to point 23 of the revised Declaration of Helsinki from 2008, this study was approved by the local ethics committee of Ludwig-Maximilians-University of Munich (approval number 554-11, 23 January 2012). All patients included in the clinical studies gave written informed consent before any study-specific procedure was performed; patients included in our local prospective biomarker study provided informed consent for the use of their clinical data and archival tumor tissue for scientific research.

### 4.2. Tumor Samples, SMAD4 Immunohistochemistry and Scoring Algorithm

We retrieved formalin fixed paraffin embedded (FFPE) archival tumor tissue of 143 PC patients irrespective of the tissue origin (primary or metastatic, resection or biopsy). To examine tumoral SMAD4 expression using IHC on 4 µm whole mount tissue sections, a Ventana Benchmark Ultra autostainer was employed (Ventana, Tucson, AZ, USA). Briefly, the slides were dewaxed and antigenicity was retrieved using the Ventana antigen retrieval solution CC1 (pH 8.4, Ventana) for 64 min. The slides were then incubated with the rabbit polyclonal anti-SMAD4 antibody (1:300 dilution, Atlas Antibodies, Stockholm, Sweden) for 32 min and after secondary antibody incubation (OptiView Kit; Ventana) the staining was visualized using a diaminobenzidine system (Ventana). Microphotographs were acquired on a camera equipped Axioskop microscope (Zeiss, Jena, Germany) using proprietary Axiovision software version 4.9 (Zeiss). Normal tissue, such as adjacent liver parenchyma in liver biopsies or pancreatic acinar tissue in resection specimens served as internal positive controls for SMAD4 expression. As the normal tissue present served for comparison to the tumor tissue, the samples in which no SMAD4 expression could be detected in adjacent normal tissue were excluded from the analysis.

The absence or presence of SMAD4 expression was assessed as previously described [19] by two experienced pathologists (SO and TK) blinded to the patients’ clinical outcome. Discrepant cases were discussed until agreement was reached. Briefly, SMAD4 was considered as “expressed” if tumor cells showed a moderate to strong cytoplasmic immunolabeling comparable to the adjacent normal tissue. SMAD4 expression was considered as “lost” when no staining at all, a very faint overall staining or a weak staining in some tumor cells compared to normal tissue was detected, or when there was no detectable SMAD4 expression in the majority of the tumor cells. The results were correlated with the patients’ previously determined clinicopathological parameters and survival times (both PFS and OS) from the beginning of palliative chemotherapy.

### 4.3. Statistical Analyses

The Kaplan–Meier method with log-rank tests and Cox regression were used for univariate survival analyses. For multivariate analysis, a stepwise forward Cox regression model was used. The relation of SMAD4 expression to the pts’ clinicopathological characteristics was evaluated using cross tabulations and χ^2^ tests.

## 5. Conclusions

Taken together, our data show that SMAD4 loss alone cannot be considered a bona fide negative prognostic biomarker in advanced PC. Patients with SMAD4-negative tumors may potentially benefit from a gemcitabine- and/or a gemcitabine–fluoropyrimidine-based palliative chemotherapy, specifically with regard to improvements in PFS. As the plethora of data on SMAD4 in resected PC is all based on heterogeneous methodology, standardized methods and cut-offs are required to draw reliable conclusions about its function as a prognostic biomarker in PC. Additionally, physicians and researchers should be aware that the same biomarker may have different implications according to the patients´ clinical situation (i.e., resectable vs. advanced PC) and ethnic (i.e., genetic) background.

## Figures and Tables

**Table 1:** Frequency of the patients’ clinicopathological characteristics (sex, age, Karnofsky performance status (KPS), disease stage at start of first-line chemotherapy, type of first-line chemotherapy (CTX), tumor grade and number of metastatic sites) and correlation to overall survival (OS) and progression-free survival (PFS) with corresponding hazard ratios (HR) and confidence intervals (CI).

Characteristics	Group	*n*	OS (Months)	*p* (Log-Rank)	HR	95% CI	PFS (Months)	*p* (Log-Rank)	HR	95% CI
Sex	male	84	8.3	0.673	0.926	0.649–1.323	6.8	0.578	1.123	0.745–1.692
	female	59	8.3				5.3			
Age group	<60 years	55	8.3	0.414	1163	0.810–1.670	6.4	0.859	1.038	0.686–1.571
	≥60 years	88	8.4				6.7			
KPS	≤80	57	6.3	0.017	1566	1.081–2.280	4.1	0.056	1.511	0.987–2.314
	>80	76	11.1				7.6			
Stage at start of palliative CTX	locally advanced	15	13.6	0.064	1782	0.957–3.317	10.0	0.054	2.015	0.972–4.177
	metastatic	128	8.1				6.3			
CTX type	gemcitabine-based	99	8.3	0.250	1091	0.863–1.379	7.6	0.017	1.336	1.040–1.715
	fluoropyrimidine-based	25	9.1				4.0			
	gem/fluo—based	19	11.0				4.7			
Tumor grade	G1–G2	61	11.5	0.171	1280	0.897–1.827	8.6	0.135	1.367	0.905–2.066
	G3–G4	82	7.8				5.5			
Metastatic sites	0	15	13.6	0.180	1208	0.908–1.608	10.0	0.097	1,439	1.022–2.025
	1	90	8.3				6.6			
	>1	38	7.8				4.7			

**Table 2:** Correlation of overall and progression-free survival (Kaplan–Meier estimates, log-rank tests and Cox regression) according to SMAD4 expression status (expressed vs. lost) in the total study population, the gemcitabine-based, fluoropyrimidine-based and gemcitabine–fluoropyrimidine-based treatment subgroups.

Chemotherapy Subgroup	SMAD4	*n*	%	OS (Months)	*p* (Log-Rank)	HR	95% CI	PFS (Months)	*p* (Log-Rank)	HR	95% CI
Total	expressed	51	35.7	7.8	0.656	1088	0.751–1.576	5.8	0.038	1565	1.020–2.399
	lost	92	64.3	8.5				7.0			
Gemcitabine	expressed	35	35.4	8.3	0.722	1069	0.680–1.679	6.8	0.037	1790	1.028–3.116
	lost	64	64.6	8.3				8.9			
Fluopyrimidine	expressed	10	40.0	11.5	0.104	0.471	0.187–1.190	3.6	0.185	0.524	0.198–1.387
	lost	15	60.0	7.2				6.7			
Gemcitabine + fluopyrimidine	expressed	6	31.6	4.3	0.017	4277	1.185–15.431	2.5	0.018	3489	1.148–10.603
	lost	13	68.4	12.5				6.2			

## Supplementary Materials

Supplementary materials can be found at www.mdpi.com/1422-0067/18/5/1094/s1.

## Abbreviations

CI	Confidence interval
DPC4	Deleted in pancreatic cancer 4
FFPE	Formalin fixed paraffin embedded
FOLFIRINOX	5-FU, folinic acid, irinotecan, oxaliplatin
HR	Hazard ratio
IHC	Immunohistochemistry
KPS	Karnofsky performance status
LAPC	Locally advanced pancreatic cancer
µm	Micrometer
nab-paclitaxel	Nanoparticle albumin-bound paclitaxel
OS	Overall survival
PC	Pancreatic cancer
PFS	Progression-free survival
pts	patients
RUNX3	Runt-related transcription factor 3
SMAD4	Mothers against decapentaplegic homolog 4
US	United States
