# Supplementary material for: The Impact of SMAD4 Loss on Outcome in Patients with Advanced Pancreatic Cancer Treated with Systemic Chemotherapy
Source: Int J Mol Sci. 2017 May 19;18(5):1094. doi: 10.3390/ijms18051094 (PMC5455003; doi:10.3390/ijms18051094)
Supplement: Supplementary file 1 [file ijms-18-01094-s001.pdf]

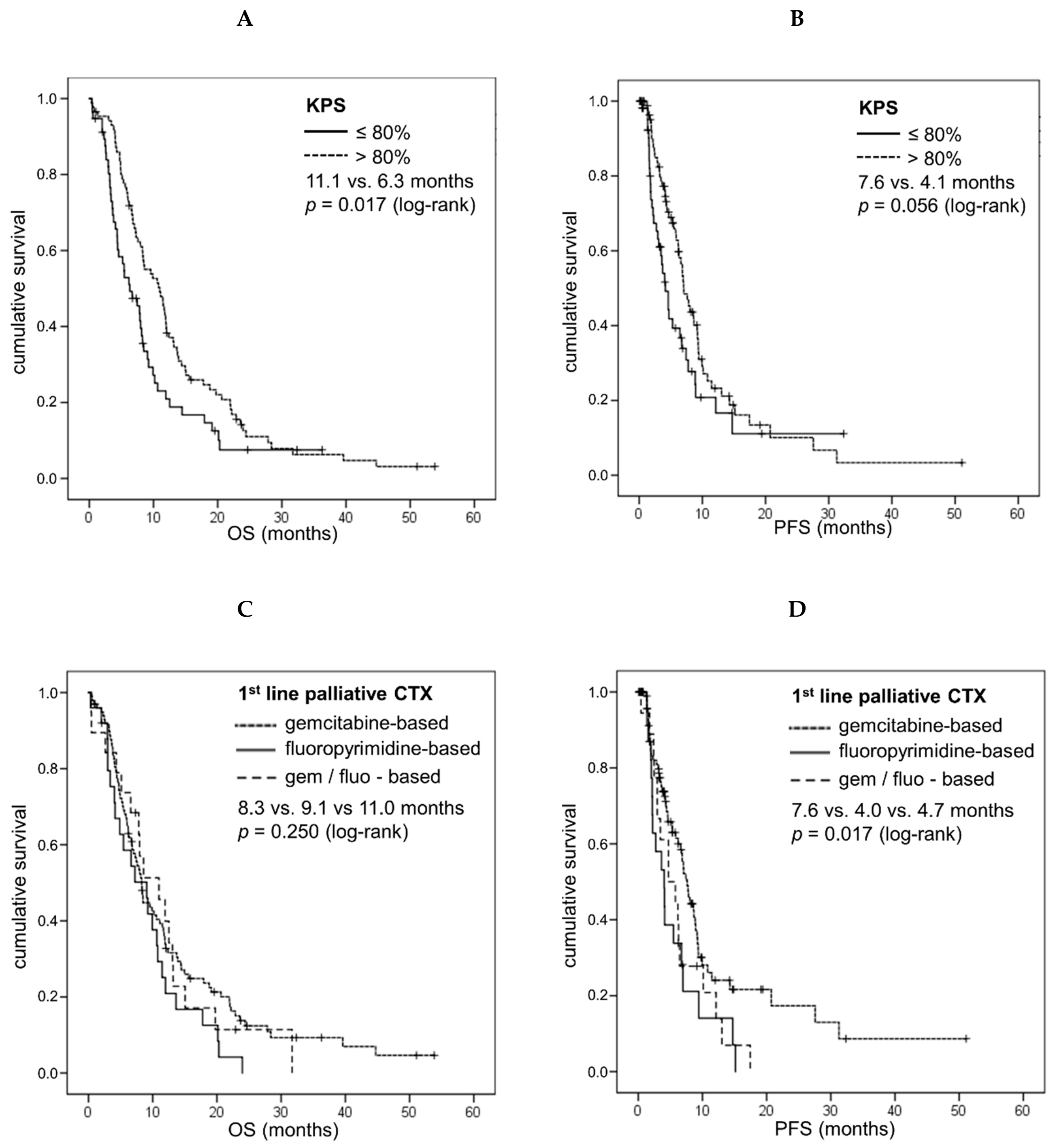

Figure S1 Univariate analysis (Kaplan–Meier curves and log-rank tests) of Karnofsky performance scale (KPS) groups (S1A, S1B) as well as type of first-line chemotherapy (CTX, S1C, S1D) and OS or PFS in palliatively-treated aPC pts. Crossed lines indicate censored cases.

Table S1 Correlation of SMAD4 expression status and pts' clinicopathological characteristics using cross tabulations and χ^2 tests.

		n	**SMAD4 Loss**	**p** (χ^2)
Sex	male	84	55	0.434
	female	59	37	
Age group	< 60 years	55	38	0.225
	≥ 60 years	88	54	
KPS	≤ 80	57	35	0.337
	> 80	86	57	
Stage at start of palliative CTX	locally advanced	15	11	0.321
	metastatic	128	81	
Tumor grade	G1 - G2	61	42	0.213
	G3 - G4	82	32	
Metastatic sites	0	15	11	0.155
	1	90	60	
	>1	38	21	
